# Case report: Subutaneous hemangiomatosis causing Kasabach-Merritt syndrome – MRI features

**DOI:** 10.4103/0971-3026.40957

**Published:** 2008-11

**Authors:** Tarun P Jain, Raju Sharma, Rohini Gupta

**Affiliations:** Department of Radiology, All India Institute of Medical Sciences, New Delhi - 110 029, India

**Keywords:** Kasabach-Merritt syndrome, MRI, hemangiomas

## Abstract

Hemangiomatosis is an uncommon entity in which there is diffuse infiltration of soft tissue or bone by hemangioma. Kasabach-Merritt syndrome is an uncommon complication of large hemangiomas, in which there is thrombocytopenia and coagulopathy. Plain radiographs, in addition to showing a soft tissue mass, also show a variety of findings in the bones. MRI is the investigation of choice. A case of a 2-year-old child suffering from hemangiomatosis and a resultant Kasabach-Merritt syndrome is presented.

Hemangioma is a commonly encountered benign vascular neoplasm. It is the most common soft tissue neoplasm of infancy.[1] Diffuse infiltration of soft tissue or bone occurs with hemangiomatosis. MRI is the modality of choice in patients with hemangiomas. Kasabach-Merritt syndrome (KMS) is a rare complication of large hemangiomas, in which the patient presents with thrombocytopenia and coagulopathy. We present the radiological features of a young child suffering from hemangiomatosis and KMS.

## Case Report

A 2-year-old female child presented with a history of easy bruisability. In addition, the parents had noticed a diffuse swelling over her chest and shoulders since birth; the swelling had not increased in size and there was no history of bleeding from it. On physical examination, a large ecchymotic patch was seen overlying the swelling; it was soft and there was no bruit. Hematological investigations showed thrombocytopenia (platelet count - 75,000/mm^3^). The blood counts were otherwise unremarkable.

**Figure 1:**
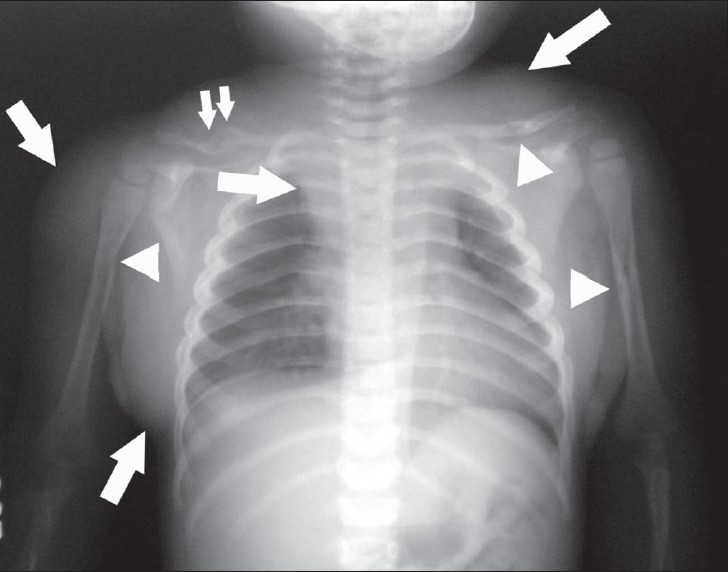
Plain radiograph of the chest and abdomen shows increased soft tissue over the shoulder and chest wall (arrows) with widening of the superior mediastinum (arrows). Cortical erosions and osteolytic areas are seen in the diaphyses of the humeri, left clavicle, and the right scapular spine (arrowheads). A healed fracture is seen in the right clavicle (double arrow)

A plain radiograph of the chest and abdomen [Figure 1] showed diffuse soft tissue swelling over the shoulder and chest wall. In addition, there were cortical erosions and medullary osteolytic areas in the diaphyses of the humeri, left clavicle, and the right scapular spine. A healed fracture was noted in the right clavicle. There was widening of the superior mediastinum. No calcifications or phleboliths were seen.

**Figure 2 (A, B):**
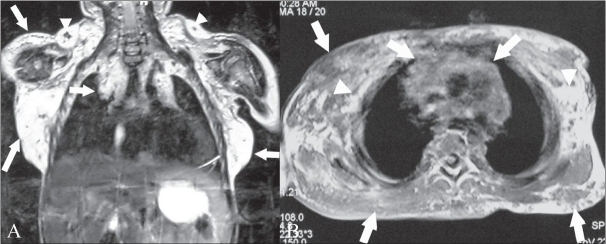
Fat-saturated, coronal T2W MRI image (A) of the chest and abdomen shows a diffusely infiltrating hyperintense lesion (arrows) in the soft tissues of the lower neck, shoulder, axillae, and chest wall, extending into the anterior mediastinum (arrows) with a few areas of focal hypointensity (arrowheads). The axial T1W MRI image (B) of the upper thorax shows the lesion to be hypointense (arrows) with interspersed fat (arrowheads)

MRI showed a diffusely infiltrating lesion in the soft tissues of the lower neck, shoulder, both axillae, and the chest wall, with extension into the anterior mediastinum. It was predominantly hyperintense on the T2W images [Figure 2a] with few focal hypointense areas, likely due to fibrous tissue or areas of thrombosis; there was variable hypointensity on T1W images with interspersed fat [Figure 2b]. In diffuse hemangiomatosis, the presence of fat throughout the lesion can be demonstrated by MRI and this helps in making this diagnosis. There was no evidence of any other lesion in the chest or abdomen.

On the basis of the above clinical, radiological, and hematological findings, a diagnosis of hemangiomatosis of the chest wall involving the mediastinum with a resultant KMS was made and the patient was prescribed steroids to reduce the size of the lesion. There was a mild reduction in the size of the lesion after initial therapy; unfortunately, the patient was later lost to follow-up.

## Discussion

Hemangiomas are the most frequently encountered vascular soft tissue abnormality.[2] They constitute 7% of all benign soft tissue tumors.[3] They are the most common soft tissue neoplasm of infancy.[1] Hemangiomas arise in a variety of locations, including the skin, subcutaneous tissue, muscle, and synovium.

On radiographs [Figure 1], an indeterminate soft tissue mass is seen which may show phleboliths. Osseous changes may also be seen, which can be periosteal (benign or aggressive periosteal reaction); cortical (erosion, thickening, tunneling, or osteopenia); or medullary (osteopenia or sclerosis) in location, correlating with the proximity of the hemangiomas to the adjacent bones.[4] USG with Doppler can show the lesions well. High vessel density and high peak arterial Doppler shift can be used to distinguish hemangiomas from other soft tissue masses.[5] USG, when used with Doppler, can also help to distinguish hemangiomas from vascular malformations.[1] CT scans show a soft tissue mass with variable enhancement and phleboliths.

MRI is the modality of choice for demonstrating the relationships between hemangiomas and the adjacent anatomic structures. Hemangiomas appear as ill-defined hyperintense masses on T2W images [Figure 2] because of the presence of cavernous or cystic vascular spaces containing stagnant blood. Fluid–fluid levels or low-signal-intensity areas (corresponding to fibrous tissue, fast flow within vessels, foci of calcification, or areas of thrombosis) may also be seen.[2] On T1W images, the lesions display a signal intensity intermediate between that of muscle and fat. Dynamic post-contrast MRI can help differentiate hemangiomas from other vascular malformations and to distinguish between low- and high-perfusion lesions, further aiding in management.[2] The lesion's morphology, signal intensity, and gadolinium enhancement allow differentiation of hemangiomas from malignant soft tissue masses.[6]

Angiomatosis is defined as diffuse infiltration of bone or soft tissue by hemangiomatous or lymphangiomatous lesions.[7] The lesion in our patient showed diffuse infiltration from the lower neck to the upper mediastinum and chest wall. The MRI appearance of angiomatosis is similar to that of solitary angiomatous lesions[2] and, in addition, there is presence of interspersed fat throughout the the lesion.

KMS is an uncommon complication of large hemangiomas, in which there is thrombocytopenia and purpura. It is a coagulopathy consisting of intravascular coagulation, clotting, and fibrinolysis within the hemangioma.[2]

The goal of therapy in a patient with hemangiomatosis is to achieve involution of the lesions, usually with corticosteroids. Surgical resection, radiation therapy, laser therapy, and angiogenesis inhibitors such as interferon are other therapeutic options.[8]

Our patient had an uncommon manifestation of diffuse hemangiomatosis with an unusual complication: the Kasabach-Merritt syndrome. MRI was able to make the diagnosis of hemangiomatosis with ease, while the clinical features helped in diagnosing the associated complication.
